# Predicting survival outcomes using subsets of significant genes in prognostic marker studies with microarrays

**DOI:** 10.1186/1471-2105-7-156

**Published:** 2006-03-20

**Authors:** Shigeyuki Matsui

**Affiliations:** 1 Department of Pharmacoepidemiology, School of Public Health, Kyoto University, Yoshida Konoe-cho, Sakyo-ku, Kyoto 606-8501, Japan; 2 Translational Research Informatics Center, Foundation for Biomedical Research and Innovation, Minatojima-minami-machi, Chuo-ku, Kobe 650-0047, Japan

## Abstract

**Background:**

Genetic markers hold great promise for refining our ability to establish precise prognostic prediction for diseases. The development of comprehensive gene expression microarray technology has allowed the selection of relevant marker genes from a large pool of candidate genes in early-phased, developmental prognostic marker studies. The primary analytical task in such studies is to select a small fraction of relevant genes, typically from a list of significant genes, for further investigation in subsequent studies.

**Results:**

We develop a methodology for predicting survival outcomes using subsets of significant genes in prognostic marker studies with microarrays. Key components in this methodology include building prediction models, assessing predictive performance of prediction models, and assessing significance of prediction results. As particular specifications, we assume Cox proportional hazard models with a compound covariate. For assessing predictive accuracy, we propose to use the cross-validated log partial likelihood. To assess significance of prediction results, we apply permutation procedures in cross-validated prediction. As an additional key component peculiar to prognostic prediction, we also consider incorporation of standard prognostic factors. The methodology is evaluated using both simulated and real data.

**Conclusion:**

The developed methodology for prognostic prediction using a subset of significant genes can provide new insights based on predictive capability, possibly incorporating standard prognostic factors, in selecting a fraction of relevant genes for subsequent studies.

## Background

Genetic markers hold great promise for refining our ability to establish precise prognostic prediction for diseases. The development of comprehensive, gene expression microarray technology has allowed the selection of relevant marker genes from a large pool of candidate genes in early-phased, developmental prognostic marker studies for various cancers including diffuse large B-cell lymphoma [1], follicular lymphoma [2], acute myeloid leukemia [3], lung adenocarcinoma [4], and metastatic kidney cancer [5]. The selected genes will be further investigated in subsequent studies using technically simpler, but more reliable assays such as multiplexed quantitative reverse-transcriptase polymerase chain reaction (RT-PCR) in formalin-fixed, paraffin-embedded tissue sections for routine clinical use [6,7]. Accordingly, the primary task in early-phased prognostic marker studies with microarrays would be to select a small fraction of relevant genes for subsequent studies. To this end, multiple testing to identify genes associated with prognosis is typically adopted as primary analysis, which may provide a list of significant genes.

Prediction analysis using subsets of significant genes may supplement the primary analysis. It can provide information regarding predictive capability for subsets of significant genes. More importantly, provided that appropriate measures of predictive accuracy for survival outcomes are established, it may indicate another 'cut-off' for a list of significant genes on the basis of predictive accuracy through *gene filtering *other than the criteria to control false positives in multiple testing. Despites many methods for prediction analysis of survival outcomes proposed in bioinformatics and biostatistics literature, including application of partial least squares [8-10] and ridge regression [11,12], most methods intend to use the full set of genes for prediction without regard to the primary analysis.

In this article, we develop a methodology for predicting survival outcomes using subsets of significant genes. Key components in this methodology include building prediction models, assessing predictive performance of prediction models, and assessing significance of prediction results. Here, given the first two components, we can perform gene filtering. For each component, we consider particular specifications or procedures to illustrate the methodology. In building prediction models, we assume Cox proportional hazard models with a compound covariate [13,14]. In assessing performance of prediction models, measures of explained variation for Cox regression models [15-17] may not aim to measure the performance of prediction models on future patients, i.e., predictive accuracy. We propose to use the cross-validated log partial likelihood [18,19] to measure predictive accuracy. To assess significance of prediction results, we apply the permutation procedures in cross-validated prediction proposed by Radmacher et al. [14]. As an additional key component peculiar to prognostic prediction, we also consider incorporation of standard prognostic factors, because it is important to determine whether a new genetic marker adds prognostic information to that already contained in the more established prognostic factors [20]. The performance of the methodology will be evaluated using simulated data and real data from a lymphoma study.

## Results

### Gene filtering

The simplest approach of gene filtering is based on the marginal association between each gene expression and survival time [1-5]. For patient *i *in the training set, let *h*_*i*_(*t*) be the hazard function and *x*_*j*,*i *_be the expression level for gene *j*. For gene *j*, we assume the univariate Cox regression model,

*h*_*i*_(*t*) = *h*_*j*,0_(*t*) exp(*β*_*j*_*x*_*j*,*i*_)     (1)

where *h*_*j*,0_(*t*) is the baseline hazard function and *β*_*j *_is a parameter. Gene filtering is based on a test of hypothesis *β*_*j *_= 0 (e.g., a score or Wald test [21]). Genes are typically ranked on the basis of the value of absolute standardized test statistic. Gene filtering can be based on the number of genes [4] or a *P*-value cut-off [1,2,5]. A standardized score or Wald test statistic for testing hypothesis *β*_*j *_= 0 is asymptotically normal with unit variance and mean equal to *D*^1/2^*β*_*j*_*σ*_*j *_where σj2
 MathType@MTEF@5@5@+=feaafiart1ev1aaatCvAUfKttLearuWrP9MDH5MBPbIqV92AaeXatLxBI9gBaebbnrfifHhDYfgasaacH8akY=wiFfYdH8Gipec8Eeeu0xXdbba9frFj0=OqFfea0dXdd9vqai=hGuQ8kuc9pgc9s8qqaq=dirpe0xb9q8qiLsFr0=vr0=vr0dc8meaabaqaciaacaGaaeqabaqabeGadaaakeaacqaHdpWCdaqhaaWcbaGaemOAaOgabaGaeGOmaidaaaaa@30EB@ is the variance of expression levels across patients for gene *j *and *D *is the expected number of events [22]. The gene filtering is thus based on the hazard ratio associated with a change of standard deviation in gene expression for a given number of events.

### Prediction model

For the set of *K *selected genes (*j*_1_, ..., *j*_*K*_), the compound covariate for patient *i *is defined as

ci=∑k=1Kzjkxjk,i     (2)
 MathType@MTEF@5@5@+=feaafiart1ev1aaatCvAUfKttLearuWrP9MDH5MBPbIqV92AaeXatLxBI9gBaebbnrfifHhDYfgasaacH8akY=wiFfYdH8Gipec8Eeeu0xXdbba9frFj0=OqFfea0dXdd9vqai=hGuQ8kuc9pgc9s8qqaq=dirpe0xb9q8qiLsFr0=vr0=vr0dc8meaabaqaciaacaGaaeqabaqabeGadaaakeaacqWGJbWydaWgaaWcbaGaemyAaKgabeaakiabg2da9maaqahabaGaemOEaO3aaSbaaSqaaiabdQgaQnaaBaaameaacqWGRbWAaeqaaaWcbeaakiabdIha4naaBaaaleaacqWGQbGAdaWgaaadbaGaem4AaSgabeaaliabcYcaSiabdMgaPbqabaaabaGaem4AaSMaeyypa0JaeGymaedabaGaem4saSeaniabggHiLdGccaWLjaGaaCzcamaabmaabaGaeGOmaidacaGLOaGaayzkaaaaaa@4681@

where zjk
 MathType@MTEF@5@5@+=feaafiart1ev1aaatCvAUfKttLearuWrP9MDH5MBPbIqV92AaeXatLxBI9gBaebbnrfifHhDYfgasaacH8akY=wiFfYdH8Gipec8Eeeu0xXdbba9frFj0=OqFfea0dXdd9vqai=hGuQ8kuc9pgc9s8qqaq=dirpe0xb9q8qiLsFr0=vr0=vr0dc8meaabaqaciaacaGaaeqabaqabeGadaaakeaacqWG6bGEdaWgaaWcbaGaemOAaO2aaSbaaWqaaiabdUgaRbqabaaaleqaaaaa@3149@ is the standardized test statistic obtained in the gene filtering for the selected gene *j*_*k *_(*k *= 1, ..., *K*). The definition of the compound covariates weights by means of standardized test statistics has been suggested for generalized linear models in Radmacher et al. [14]. This weighting policy reflects the criterion in the gene filtering step. Another possible policy is to use an estimate of *β*_*j*_, in stead of *z*_*j*_, as the weight for gene *j *(e.g., Beer et al. [4]). Our weighting policy gives higher weight to genes with larger variance, which would yield a more robust predictor for subsequent validation studies because the expression profiles for genes with larger variance would be more reproducible.

The compound covariate can be regarded as a prognostic index; patients with large values of the compound covariate may have poor prognosis. We assume the following Cox model to relate the compound covariate to the survival time,

*h*_*i*_(*t*) = *h*_0_(*t*) exp(*ψc*_*i*_)     (3)

where *h*_0_(*t*) is the baseline hazard function and *ψ *is a parameter. The compound covariate for a new patient with the vector of expression level (xj1*,…,xjk*)
 MathType@MTEF@5@5@+=feaafiart1ev1aaatCvAUfKttLearuWrP9MDH5MBPbIqV92AaeXatLxBI9gBaebbnrfifHhDYfgasaacH8akY=wiFfYdH8Gipec8Eeeu0xXdbba9frFj0=OqFfea0dXdd9vqai=hGuQ8kuc9pgc9s8qqaq=dirpe0xb9q8qiLsFr0=vr0=vr0dc8meaabaqaciaacaGaaeqabaqabeGadaaakeaacqGGOaakcqWG4baEdaqhaaWcbaGaemOAaO2aaSbaaWqaaiabigdaXaqabaaaleaacqGGQaGkaaGccqGGSaalcqWIMaYscqGGSaalcqWG4baEdaqhaaWcbaGaemOAaO2aaSbaaWqaaiabdUgaRbqabaaaleaacqGGQaGkaaGccqGGPaqkaaa@3BD1@ for the selected genes can be calculated by replacing xjk,i
 MathType@MTEF@5@5@+=feaafiart1ev1aaatCvAUfKttLearuWrP9MDH5MBPbIqV92AaeXatLxBI9gBaebbnrfifHhDYfgasaacH8akY=wiFfYdH8Gipec8Eeeu0xXdbba9frFj0=OqFfea0dXdd9vqai=hGuQ8kuc9pgc9s8qqaq=dirpe0xb9q8qiLsFr0=vr0=vr0dc8meaabaqaciaacaGaaeqabaqabeGadaaakeaacqWG4baEdaWgaaWcbaGaemOAaO2aaSbaaWqaaiabdUgaRbqabaWccqGGSaalcqWGPbqAaeqaaaaa@3380@ with xjk*
 MathType@MTEF@5@5@+=feaafiart1ev1aaatCvAUfKttLearuWrP9MDH5MBPbIqV92AaeXatLxBI9gBaebbnrfifHhDYfgasaacH8akY=wiFfYdH8Gipec8Eeeu0xXdbba9frFj0=OqFfea0dXdd9vqai=hGuQ8kuc9pgc9s8qqaq=dirpe0xb9q8qiLsFr0=vr0=vr0dc8meaabaqaciaacaGaaeqabaqabeGadaaakeaacqWG4baEdaqhaaWcbaGaemOAaO2aaSbaaWqaaiabdUgaRbqabaaaleaacqGGQaGkaaaaaa@3222@ in (2), which is used for the prediction of survival time for that patient.

### Predictive accuracy

We use the cross-validated log partial likelihood [18,19] to measure predictive accuracy of Cox models. Specifically, the average *M*-fold cross-validated log partial likelihood is given by

ACVL=−1M∑m=1Mlm(ψ^(−m)),
 MathType@MTEF@5@5@+=feaafiart1ev1aaatCvAUfKttLearuWrP9MDH5MBPbIqV92AaeXatLxBI9gBaebbnrfifHhDYfgasaacH8akY=wiFfYdH8Gipec8Eeeu0xXdbba9frFj0=OqFfea0dXdd9vqai=hGuQ8kuc9pgc9s8qqaq=dirpe0xb9q8qiLsFr0=vr0=vr0dc8meaabaqaciaacaGaaeqabaqabeGadaaakeaacqWGbbqqcqWGdbWqcqWGwbGvcqWGmbatcqGH9aqpcqGHsisldaWcaaqaaiabigdaXaqaaiabd2eanbaadaaeWbqaaiabdYgaSnaaBaaaleaacqWGTbqBaeqaaaqaaiabd2gaTjabg2da9iabigdaXaqaaiabd2eanbqdcqGHris5aOGaeiikaGIafqiYdKNbaKaadaWgaaWcbaGaeiikaGIaeyOeI0IaemyBa0MaeiykaKcabeaakiabcMcaPiabcYcaSaaa@4787@

where *l*_*m*_(*ψ*) = *l*_*T*_(*ψ*) - *l*_(-*m*)_(*ψ*) is the difference between the partial log likelihood for the entire training set and that with the *m*th group of patients excluded as the test set, and ψ^(−m)
 MathType@MTEF@5@5@+=feaafiart1ev1aaatCvAUfKttLearuWrP9MDH5MBPbIqV92AaeXatLxBI9gBaebbnrfifHhDYfgasaacH8akY=wiFfYdH8Gipec8Eeeu0xXdbba9frFj0=OqFfea0dXdd9vqai=hGuQ8kuc9pgc9s8qqaq=dirpe0xb9q8qiLsFr0=vr0=vr0dc8meaabaqaciaacaGaaeqabaqabeGadaaakeaacuaHipqEgaqcamaaBaaaleaacqGGOaakcqGHsislcqWGTbqBcqGGPaqkaeqaaaaa@32B8@ is the value of *ψ *that maximizes *l*_(-*m*)_(*ψ*) for *m *= 1, ..., *M*. As to the number of cross-validation groups, *M *= 10 or 5 are reasonable choices especially for computationally burdensome analyses for large samples [23]. A low value of *ACVL *corresponds to high predictive accuracy. *ACVL *reduces to the prediction residual error of sum of square (PRESS) in normal linear models [18].

When using *M*-fold cross-validation, it is critical that all aspects of model building including gene filtering are re-performed for each of *M *rounds of cross-validation to avoid selection bias [24,25]. If we choose the cut-offs in gene filtering that minimizes *ACVL*, an independent validation set would be needed to have unbiased estimate of predictive accuracy because of the optimization process in model building for the training set. Matsui [26] demonstrated that the bias due to the optimization can be substantial in a class prediction problem from gene expression profiling using 6,000 genes for 48 bladder tumors.

A limitation of *AVCL *is that it is difficult to interpret for non-statisticians. Some graphical displays may be helpful to interpret the results. If an independent validation set is available, a simple way is to divide the validation set into some groups based on the value of the prognostic index and compare survival curves between groups using a log-rank test. The same type of assessment can also be performed for cross-validated test sets from the training set, but a usual log-rank test is not valid because the groups are not pre-specified independently of the survival time. A permutation procedure which permutates survival time to expression profile is available to have a correct *P*-value [14,5]. In this procedure, the same cross-validated model building process, with some optimization process such as choosing optimal cut-off based on *ACVL*, if any, is performed to permutated data to obtain a null distribution of the log-rank statistic. This permutation procedure can also be useful for assessing the statistical significance of (the minimized) *ACVL*, in which one may obtain a null distribution of (the minimized) *ACVL*.

### Adjustment for prognostic factors

Let *u*_*i *_be a vector of prognostic factors for patient *i*. For gene *j*, we assume the Cox model, instead of (1),

hi(t)=hj,0(t)exp⁡(ηj'ui+βjxj,i)     (4)
 MathType@MTEF@5@5@+=feaafiart1ev1aaatCvAUfKttLearuWrP9MDH5MBPbIqV92AaeXatLxBI9gBaebbnrfifHhDYfgasaacH8akY=wiFfYdH8Gipec8Eeeu0xXdbba9frFj0=OqFfea0dXdd9vqai=hGuQ8kuc9pgc9s8qqaq=dirpe0xb9q8qiLsFr0=vr0=vr0dc8meaabaqaciaacaGaaeqabaqabeGadaaakeaacqWGObaAdaWgaaWcbaGaemyAaKgabeaakiabcIcaOiabdsha0jabcMcaPiabg2da9iabdIgaOnaaBaaaleaacqWGQbGAcqGGSaalcqaIWaamaeqaaOGaeiikaGIaemiDaqNaeiykaKIagiyzauMaeiiEaGNaeiiCaaNaeiikaGIaeq4TdG2aa0baaSqaaiabdQgaQbqaaiabcEcaNaaakiabdwha1naaBaaaleaacqWGPbqAaeqaaOGaey4kaSIaeqOSdi2aaSbaaSqaaiabdQgaQbqabaGccqWG4baEdaWgaaWcbaGaemOAaOMaeiilaWIaemyAaKgabeaakiabcMcaPiaaxMaacaWLjaWaaeWaaeaacqaI0aanaiaawIcacaGLPaaaaaa@55BC@

where *η*_*j *_is a vector of parameters. Gene filtering is based on a test of hypothesis *β*_*j *_= 0 in model (4). This is to select genes after adjustment for the prognostic factors. For the set of *K *selected genes (*j*_1_, ..., *j*_*K*_), we calculate the compound covariate *c*_*i *_in (2) using a standardized test statistic for the hypothesis . Then we assume the Cox model, instead of (3),

*h*_*i*_(*t*) = *h*_0_(*t*) exp(*γ*'*u*_*i *_+*ψc*_*i*_)    (5)

where *γ *and *ψ *are parameters. We assess the predictive accuracy based on *ACVL *for model (5). The prediction is based on the prognostic index, γ^'ui+ψ^ci
 MathType@MTEF@5@5@+=feaafiart1ev1aaatCvAUfKttLearuWrP9MDH5MBPbIqV92AaeXatLxBI9gBaebbnrfifHhDYfgasaacH8akY=wiFfYdH8Gipec8Eeeu0xXdbba9frFj0=OqFfea0dXdd9vqai=hGuQ8kuc9pgc9s8qqaq=dirpe0xb9q8qiLsFr0=vr0=vr0dc8meaabaqaciaacaGaaeqabaqabeGadaaakeaacuaHZoWzgaqcaiabcEcaNiabdwha1naaBaaaleaacqWGPbqAaeqaaOGaey4kaSIafqiYdKNbaKaacqWGJbWydaWgaaWcbaGaemyAaKgabeaaaaa@37D3@ where  and  are estimates of *γ *and *ψ*, respectively, obtained from the training set.

Analyses should test whether new systems add predictiveness once outcome is adjusted for the effect of standard prognostic factors [20]. For the validation set, a graphical display similar to that described in the previous section may be drawn for each stratum by prognostic factors and compare survival curves using a log-rank test for each stratum or a stratified log-rank test. For cross-validated test sets, a stratum-adjusted permutation procedure would be useful, in which the observed value of the log-rank statistic or (the minimized) *ACVL *are referred to their null distribution obtained by permutating survival time to expression profile within each stratum.

### Simulated data

In this section, we assessed adequacy of choosing the cut-offs in gene filtering for the training set based on *ACVL *for the Cox model (3) through a small simulation study. We simulated data on 2,000 genes for 100 patients. Of the 2,000 genes, 50 genes were configured to be informative, i.e., these genes are associated with survival time. For informative genes, the distribution of expression was normal with mean 0 and standard deviation 1 (supposing a standardized expression data across patients for each gene). We considered exchangeable correlation matrices with correlation coefficient *ρ *of 0.2 or 0.7. In addition, we considered the correlation matrix obtained from the data from the lymphoma study by Rosenwald et al. [1] for the top 50 genes in the gene filtering with model (1). The range of correlation was -0.53 to 0.98. The informative genes were associated with survival time via a multivariate proportional hazard model,

*λ*_0_(*t*) exp(*β*'*x*)     (6)

where *λ*_0_(*t*) denotes the baseline hazard function, *β *a vector of regression parameters, and *x *a vector of gene expression for the 50 informative genes. We assumed a constant baseline hazard. We set the value of parameters to mimic the lymphoma data; the baseline hazard was set equal to 0.13 (/year) and all the elements of *β *to 0.5 (= log(1.65)), corresponding to a 1.65-fold in the hazard of failing with a standard deviation increase in gene expression. Note that the range of the absolute value of the estimate of *β *for the top 50 genes obtained from a standardized lymphoma data was 0.39 to 0.60. For parameter *β*, we also considered an estimate of *β *from the top 50 genes in the lymphoma data, which were mixture of positive and negative values, but we obtained similar results. For non-informative genes, the distribution of expression was normal with mean 0 and variance-covariance equal to the identity matrix. For each of simulated data, we calculated *ACVL *with *M *= 5 for the Cox model (3) with the compound covariate for several values of *K*. For *K *≤ 20, we also calculated *ACVL *for a multiplicative model of the form of equation (6) that included as covariates the *K *genes selected during the gene filtering step. Note that using the full set of *K *genes with *K *> 20 gave noninvertible covariance matrices in maximizing the partial likelihood, which is an inherent limitation of fitting multivariate Cox models with a number of predictors. We considered a constant censoring rate of 0.1 (/year), again, to mimic the lymphoma data. Table 1 shows the averaged *ACVL *for each of several values of *K *across 100 simulations. The *ACVL *for the model (3) was minimized in expectation when the cut-off in terms of the number of selected genes (*K*) was set equal to the number of informative genes, 50. The *ACVL *for the model (3) was smaller than that for the multivariate Cox model (6) for the top *K *(≤ 20) genes.

### Lymphoma data

We illustrated the developed methodology using the data from Rosenwald et al. [1] for diffuse large B-Cell lymphoma . Briefly, this study collected gene expression data from cDNA microarrays using pretreatment biopsy specimens and clinical data for 240 diffuse large B-Cell lymphoma patients. Clinical data included the International Prognostic Index (IPI) Score [27], which is a composite score reflecting age, tumor stage, performance status, lactate dehydrogenase level, and the number of sites of extranodal disease, before treatment as a prognostic factor. 7399 microarray features were subject to analysis for predicting the survival time after treatment. In the prediction analysis, the patients were randomly divided into two groups: the training set comprised 160 patients and the validation set comprised 80 patients. The number of events (the median survival in year) was 88 (3.9) for the training set and 50 (3.6) for the validation set.

**Figure 1:**
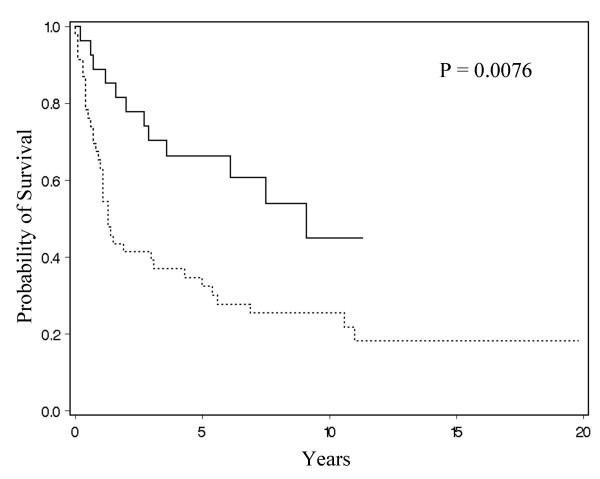
**Kaplan-Meier estimates of survival for the low IPI stratum (solid curves) and the high IPI stratum (dashed curves) in the validation set**. The *P*-value pertains to a two-sided log-rank test.

Before demonstrating our methods, we assessed the prognostic value of IPI Score. In the dataset, IPI score had three levels, low, medium, and high in terms of the risk of death. Because only 10 out of 80 patients were in the high IPI risk stratum in the validation set, we combined the medium and high IPI risk stratum into one stratum and referred to it as the high IPI risk stratum. Figure 1 shows estimated survival distributions for the low IPI risk stratum (27 patients) and the high IPI risk stratum (46 patients) in the validation set (IPI Score was missing for 7 patients), which indicates a substantial prognostic capability of IPI Score (the *P*-value of a log-rank test was 0.0076).

**Figure 2:**
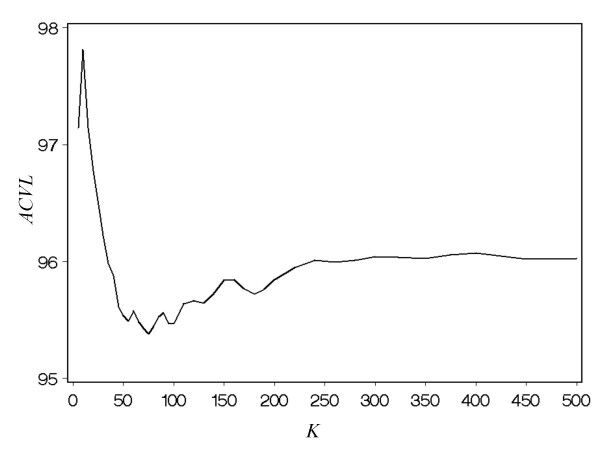
Plot of *ACVL *as *K *is varied. The *ACVL *is minimized when *K *= 75.

First we performed prediction only using gene expression data. We performed 5-fold cross-validation in building prediction models with the compound covariate for the training set. For each fold of cross-validation, we performed gene filtering from scratch to select the top *K *genes in terms of the significance level for a Wald test of *β*_*j *_= 0 for model (1) and then fitted the model (3) with the compound covariate (2) from the top *K *genes. We chose the optimal number of genes based on *ACVL *for model (3). Figure 2 shows *ACVL *for a range of *K*, indicating that *ACVL *is minimized when *K *= 75. The *P-*value for the minimized *ACVL *obtained by the permutation procedure (2,000 permutations) for the training set was less than 0.0005, which was highly significant.

**Figure 3:**
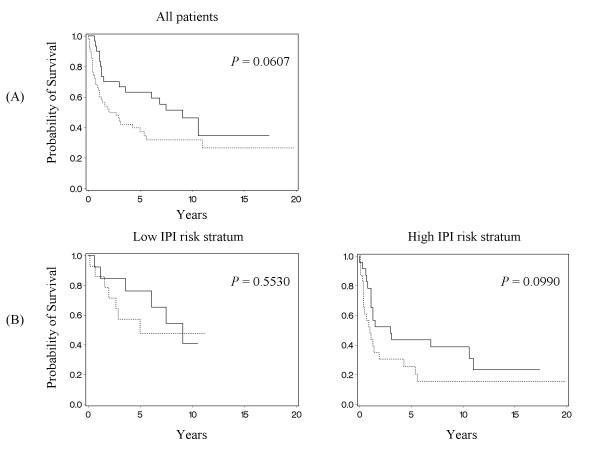
**Kaplan-Meier estimates of survival for the two groups based on the compound covariate (CC) (the low CC group (solid curves) and the high CC group (dashed curves) for all patients (Panel A) and for each stratum by IPI Score (Panel B) in the validation set**. The *P*-value in graph pertains to a two-sided log-rank test. The *P*-value for a stratified log-rank test is 0.0941.

To compare the predictive capability of the compound covariate from the 75 selected genes with that of IPI Score for the validation set, we divided the validation set into two groups based on the value of compound covariate from the 75 selected genes using the division ratio of 27: 46, which is identical with the ratio when the patients in the validation set is divided based on the two-leveled IPI Score mentioned above. Figure 3 shows estimated survival distributions for the two groups in the validation set without and with stratification by IPI Score. The 75 selected genes had a predictive capability by themselves (the *P*-value of a log-rank test was 0.0607), but it was rather weak, especially, for the low IPI risk stratum (the *P*-value of a log-rank test was 0.5530 for this stratum and the *P*-value of a stratified log-rank test was 0.0941).

**Figure 4:**
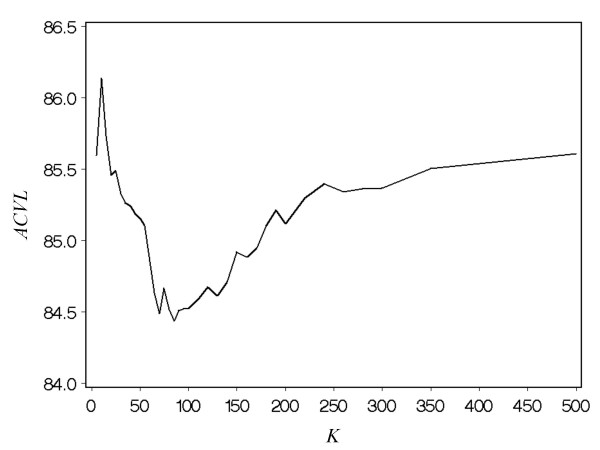
Plot of *ACVL *as *K *is varied after adjustment for IPI Score. The *ACVL *is minimized when *K *= 85.

Next, we performed gene filtering after adjustment for IPI Score and built prediction models using both IPI Score and gene expression data. This analysis would be warranted in the presence of substantial predictive capability of IPI Score as indicated by Figure 1. Again, we performed 5-fold cross-validation for the training set. Gene filtering was based on a Wald test of *β*_*j *_= 0 for model (4) with *u*_*i *_for IPI Score (*u*_*i *_= 1 if IPI Score is high, and *u*_*i *_= 0 otherwise). Figure 4 shows *ACVL *for model (5) as *K *is varied, indicating that *ACVL *is minimized when *K *= 85. The *P*-value from the stratum-adjusted permutation procedure (2,000 permutations) to assess statistical significance of the minimized *ACVL *after adjustment for IPI Score was 0.024, which was still significant at the significance level of 0.05. This means that gene expression data have additional prognostic information independent of IPI Score.

**Figure 5:**
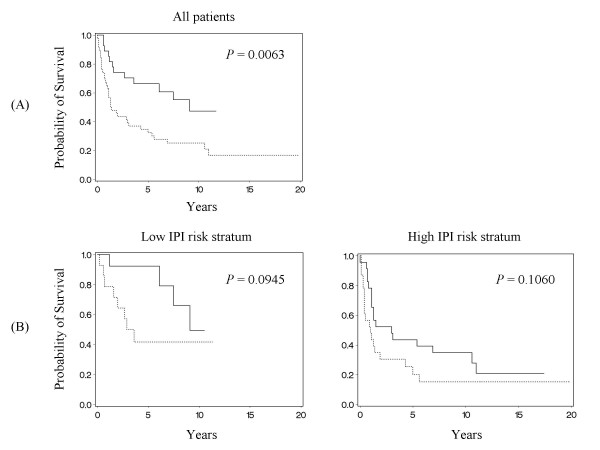
**Kaplan-Meier estimates of survival for the two groups based on the prognostic index (the low prognostic index group (solid curves) and the high prognostic index group (dashed curves) for all patients (Panel A) and for each stratum by IPI Score (Panel B) in the validation set**. The *P*-value in graph pertains to a two-sided log-rank test. The *P*-value for a stratified log-rank test is 0.0293.

For the validation set, Figure 5 shows estimated survival distributions between the two groups based on the prognostic index without and with stratification by IPI Score. The predictive capability of both IPI Score and the 85 selected genes was substantial (the *P*-value of a log-rank test was 0.0063). The predictive capability for the IPI low risk stratum was improved (the *P*-value of a log-rank test was 0.0945 for this stratum and the *P*-value of a stratified log-rank test was 0.0293).

Table 2 summarizes the selected genes by gene-expression signatures. 43 genes were selected from both the gene filtering with no adjustment for IPI Score and that with the adjustment (Group I). 32 genes were selected from the gene filtering with no adjustment, but not selected from that with adjustment (Group II). 42 genes were not selected from the gene filtering with no adjustment, but selected from that with adjustment (Group III). It is interesting that Group III contains 7 genes from major histocompatibility complex (MHC) class II, while Group II contains only one gene from this gene-expression signature. The number of differential expressed genes evaluated by a two-sample *t*-test to compare expressions between the two levels of IPI Score at the significance level of 0.05 was 9 (21%), 16 (50%), and 0 (0%), for Group I, II, and III, respectively. Group III has a potential to be genetic markers independent of IPI Score.

## Discussion

In this article, we have developed a methodology for predicting survival outcomes using subsets of significant genes in early-phased, developmental prognostic marker studies with DNA microarrays. Key components in this methodology include development of prediction models, assessment of predictive capability of prediction models, and assessment of significance of prediction results. To illustrate the methodology, we introduced a particular prediction model, Cox regression models with a compound covariate, and a particular measure of predictive accuracy, *ACVL*. Although adequacy of them was indicated through their application to simulated data and real data, further studies to evaluate their performance through comparison with other specifications or methods would be warranted.

With respect to specification of prediction model, Bair and Tibshirani [28] recently developed a semi-supervised method that adopted principal components analysis for developing a compound index using subsets of significant genes from the supervised, univariate Cox regression analysis with model (1). They used the first principal component, in stead of the compound covariate *c*_*i *_in (2), as a single covariate in the Cox model (3). The semi-supervised method using subsets of significant genes performed well compared with various methods using a combination of all of the genes for some cancer datasets and simulated datasets. More recently, Li and Gui [10] considered the application of partial least square and proposed multivariate Cox regression models using the first few orthogonal compound covariates for a full set of genes. The use of the second or higher orthogonal compound covariates proposed by Li and Gui, in addition to the first compound covariate like *c*_*i *_in (2), for subsets of significant genes has the potential to improve predictive accuracy. A comparison study for several prediction methods including those described above using subsets of significant genes is ongoing and the result will appear in a future report. Our method with the compound covariate *c*_*i *_in (2) and the method proposed by Li and Gui using subsets of significant genes are expected to perform well because they are purely supervised. One potential drawback for applying principal components and partial least squares in practice is that they need a complete expression dataset with no missing values for the set of *K *selected genes. Because there is generally a large number of missing values in the dataset, a complete case analysis [29] will entail a substantial efficiency loss. As such, these methods may necessitate a data imputation step prior to model fitting. Meanwhile, the univariate standardized test statistics as the weights in compound covariate *c*_*i *_in (2) can be calculated using all the observed expression levels for the set of *K *selected genes, i.e., an available data analysis [29] can be performed.

As to measure of predictive accuracy for survival outcomes, methods based on the time-dependent ROC curves and area under the curves [30] can be useful for microarray data [10].

The significance of integrating gene expression profiling into prognostic prediction studies is to improve the predictive capability attainable only using standard prognostic factors. However, it is rare that prognostic factors are incorporated in the prediction analysis using gene expression data in the literature. As an additional key component of our methodology, we considered selection of relevant genes with the adjustment for prognostic factors. The selected genes have the potential to be genetic markers unrelated to the prognostic factors. In such analysis, it is crucial to demonstrate additional information gain from genetic markers. We provided ways to assess this gain both for an independent validation set and cross-validated test sets. Comparison of the selected genes between without and with adjustment for prognostic factors would provide some insights in understanding biological mechanisms in the disease progression and help determine a set of genes for further investigation in subsequent studies. It is advisable that the comparison is supplemented by analyses of differentially expressed genes across different levels of prognostic factors.

## Conclusion

We develop a methodology for predicting survival outcomes using a subset of significant genes in prognostic marker studies with microarrays. The adequacy of the methodology was demonstrated through its application to simulated and real data. Our methodology can provide new insights based on predictive capability, possibly incorporating standard prognostic factors, in selecting a fraction of relevant genes for subsequent studies.

## Figures and Tables

**Table 1:** The averaged *ACVL *for several values of *K *across 100 simulations. Of the 2,000 genes, 50 genes were configured to be informative. The number of patients was 100.

	Correlation matrix					
	
	Exchangeable				Lymphoma data*	
			
	*ρ *= 0.2		*ρ *= 0.7			
			
*K*	CC^†^	MC^‡^	CC	MC	CC	MC
1	41.2	41.2	51.6	51.6	48.2	48.2
5	33.3	34.1	46.7	48.2	45.6	46.4
10	29.3	33.2	43.3	46.6	45.2	48.2
20	24.9	49.0	38.4	62.1	45.0	57.4
50	17.3	-	34.6	-	41.7	-
75	21.3	-	40.1	-	42.3	-
100	25.3	-	44.0	-	43.2	-
200	36.0	-	53.3	-	47.0	-
500	50.6	-	63.6	-	53.9	-
1000	56.6	-	70.3	-	58.8	-

*The correlation matrix obtained from the lymphoma data for the top 50 genes in the gene filtering with model (1).

^†^Cox model with the compound covariate (model (3))

^‡^Multivariate Cox model (model (6)) for the top *K *(≤ 20) genes

**Table 2:** The selected genes from the gene filtering with no adjustment for IPI Score and from that with the adjustment by gene-expression signature.

Group	Gene filtering		Gene class				
		
	No Adjust.	Adjust.	MHC-II	Proliferation	Lymph-node	Unknown	Total
I	Selected	Selected	16	20	1	6	43
II	Selected	Not Selected	1	20	0	11	32
III	Not Selected	Selected	7	23	0	12	42
